# α-Ketoglutarate Alleviates the Pathogenesis of Systemic Lupus Erythematosus via Reducing Oxidative Stress and Attenuating Excessive Immune Response

**DOI:** 10.3390/nu18213600

**Published:** 2026-10-30

**Authors:** Yuting Ji, Jie Li, Zhiyao Zhao, Zhenglong Gu

**Affiliations:** 1 Greater Bay Area Institute of Precision Medicine (Guangzhou), School of Life Sciences, Fudan University, Guangzhou 511458, China; 2 Bioscience and Biomedical Engineering Thrust, Systems Hub, The Hong Kong University of Science and Technology (Guangzhou), Guangzhou 511453, China; 3 Department of Nutrition and Health, China Agricultural University, Beijing 100193, China

**Keywords:** α-ketoglutarate (AKG), Systemic Lupus Erythematosus (SLE), antioxidation, mitochondrial respiration, inflammation

## Abstract

Background: Systemic lupus erythematosus (SLE) is a systemic autoimmune disease driven by dysregulated immune activation, with heterogeneous clinical manifestations and multi-organ injury. α-ketoglutarate (AKG) is a key intermediate metabolite of the tricarboxylic-acid cycle (TCA), participating in various physiological processes, including energy metabolism and immune regulation. Methods: In this study, 8-week-old female BALB/c mice were injected with pristane to establish an SLE mouse model. Using this model, we explored the therapeutic function and underlying mechanism of AKG intervention on SLE. Results: AKG administration significantly reduced serum antinuclear antibody (ANA) levels and alleviated renal injuries and biochemical improvement in SLE mice. Furthermore, it effectively maintained glomerular integrity and decreased the serum levels of biochemical enzymes and indices. Meanwhile, oxidative stress was ameliorated associated with the capacity of endogenous antioxidant enzymes and reducing the level of malondialdehyde (MDA). Most importantly, AKG upregulated the transcriptional levels of genes related to mitochondrial respiratory-chain complex, and increasing the production of adenosine triphosphate (ATP) in kidney tissues. Moreover, AKG administration regulated the proportion of spleen immune cells, and improved inflammation by decreasing the Interferon-stimulated genes (ISGs) expression levels, suppressing the liberation of inflammatory cytokines, ultimately alleviating SLE-associated damage. Conclusions: In summary, AKG protects against SLE through multi-targeted pathways, including suppressing oxidative stress, improving mitochondrial biogenesis-related factors, modulating immune-cell profiles, and dampening inflammatory response. These findings indicate that AKG has the effect of improving SLE.

## 1. Introduction

Systemic lupus erythematosus (SLE) is conventionally defined as a persistent autoimmune disorder with heterogeneous clinical manifestations affecting the skin, kidneys, brain, blood vessels, and joints [1,2,3]. SLE occurs about 3.4 million people globally, about 400,000 new patients being diagnosed every year [4,5]. It predominantly affects women in puberty and menopause [6]. According to a 2023 worldwide prevalence study of SLE, the highest incidence was reported in Poland, the United States, Barbados, and China [4]. Although the etiology remains unclear, the risk of suffering SLE is considered to be influenced by hereditary factors, mitochondrial dysfunction [7], epigenetic alterations, environmental triggers, and hormonal influences [8,9].

Accumulating evidence highlights the role of mitochondrial dysfunction and cellular oxidative stress, which can contribute to SLE pathogenesis. Previous studies have shown that enhanced mitochondrial metabolism was crucial for immune-cell proliferation and function [10]. The balance between oxidative phosphorylation (OXPHOS) and the tricarboxylic-acid cycle (TCA) maintains cellular-energy metabolism, while serving as the dynamic regulatory center for the generation and clearance of reactive oxygen species (ROS) to sustain cellular redox homeostasis [11]. Meanwhile, persistent oxidative stress disrupts mitochondrial integrity, promotes the release of mitochondrial DNA (mtDNA) into the cytoplasm, activates the cyclic GMP-AMP synthase-stimulator of interferon genes pathway (cGAS-STING), and enhances the type I interferon response [12].

Abnormal B lymphocyte expansion and immune activation play a critical role in the development of SLE [13]. SLE is characterized by increased production of autoantibodies such as ANA, anti-Sm, and anti-double-stranded DNA (anti-dsDNA) antibodies [1,4]. These autoantibodies are generated by plasma cells and terminally differentiated B cells (plasmablasts) [14]. These antibodies form immune complexes (ICs), which cause widespread organ involvement to induce local inflammation [15,16]. Furthermore, the ICs amplify the immune system and induce pro-inflammatory mediators such as IFNα, by triggering downstream signals to aggravate lupus pathology [2]. A well-documented positive correlation exists between IFN-I and SLE. Indeed, the contents of IFN-I in blood are increased in about 50% of SLE patients [17]. A larger proportion of SLE patients are estimated to carry overexpressed levels of IFN-I signaling-related genes in peripheral blood cells [18,19,20].

α-ketoglutarate (AKG) is a crucial metabolite in the TCA cycle, where it performs essential functions [21]. It has been shown that AKG plays an important role in antiviral, antitumor, and anti-inflammatory systems, by modulating the activation and differentiation of immune cells [22,23,24]. Notably, AKG could upregulate the expression of ENTPD1 and suppress the activation and differentiation of B cells by increasing the content of CD39, thereby significantly alleviating the pathogenesis of the MRL/lpr lupus model [24]. AKG can be naturally synthesized in humans and mice, and the safety and edibility have been recognized in regulatory agencies [25]. It positively affects various tissues, including the kidney, spleen, liver, heart, and so on [21]. Furthermore, AKG has protective abilities in preventing mitochondrial dysfunction and oxidative stress [26]. As research evidence has confirmed, AKG confers prominent therapeutic efficacy in ameliorating autoimmune-associated disorders, effectively improving rosacea by activating the OXGR1-Gq-MYL9 signaling axis [27]. Although the beneficial effects of AKG on immune disease are evident, the mechanisms underlying the protective effects of AKG in SLE remain unclarified.

In the current study, we established a pristane-induced SLE mice model to explore the potential preventive effects of AKG. AKG was administered after pristane induction for 3 months, and its effects on SLE were systematically evaluated. We assessed kidney structure and function, oxidative-stress response, mitochondrial biogenesis-related factors, and inflammatory responses. Analyses were conducted at the histopathological, serological, and gene transcription levels to explore the effect of AKG against the SLE mice model and to clarify its mechanisms, aiming to provide a novel candidate for clinical application. Notably, there is just a single dose in this study, clinical translation will require further validation, including dose-response and safety studies before any clinical experiment.

## 2. Materials and Methods

### 2.1. Chemicals and Reagents

Pristane (Pris; synthetic, ≥98% GC) was obtained from Sigma-Aldrich (Shanghai, China) Trading Co., Ltd. (Cat. No. D8740; CAS: 1921-70-6; Shanghai, China). α-ketoglutarate (AKG; purity ≥ 98%, determined by NaOH titration method) was bought from Sigma-Aldrich (Shanghai, China) Trading Co., Ltd. (Cat. No. K1750; CAS: 328-50-7; Shanghai, China).

### 2.2. Animals

Female BALB/c mice of equal weight, aged 8 weeks, were procured from Charles River Laboratories (Guangzhou, China). Prior to the experiment, animals were acclimatized to the laboratory environment for at least seven days. All animals were housed in a controlled room with constant conditions (animal illumination: 15–20 Lux; humidity: 40–70%; temperature: 20–26 °C; pressure: 45 Pa; light: 12 h/12 h light/dark cycle) and provided free access to food and drinking water. The animal experimental design follows the “Guide for the Care and Use of Laboratory Animals”, and all study procedures were approved by the Committee on the Ethics of Animal Experiments of Greater Bay Area Institute of Precision Medicine (Guangzhou) (Approval Code: IPMGBA-24-0018, Approval Date: 13 March 2024).

### 2.3. Experimental Design

At first, 56 female BALB/c mice were randomly assigned to two groups. One of them was intraperitoneally (i.p.) injected with 500 μL pristane to establish a systemic lupus erythematosus (SLE) disease model [28], while the other group was injected with a matching volume of phosphate-buffered saline (PBS) as healthy control. After injection, the mice were cultured for 5 months, serum was collected by capillary blood sampling and urine was also obtained to confirm the successful establishment of the SLE model. Then, mice were treated by gavage with α-ketoglutarate (AKG, 100 mg/kg/day) and an equal volume of water as a control for 3 months. The AKG dose was identified based on previous reports [24,29,30], and the preliminary experiments confirmed the safety of this concentration. Finally, the mice were randomly divided into four groups: injected with PBS and given water gavage (Control), injected with PBS and given AKG gavage (AKG), injected with pristane and given water gavage (Pris), and injected with pristane and given AKG gavage (Pris + AKG), with 14 mice in each group (*n* = 14). During the experiment, the health conditions, survival rate, and body weight of the mice are recorded. Multiple mice in the Pris group suffered severe ascites and spontaneous deaths, and only 6 valid surviving mice successfully completed the full procedure.

A total of 14 mice were initially assigned to each group. The intervention lasted three months. Mortality occurred during the intervention period and was confined mainly to the Pris group (survived: 6/14), whereas survival was markedly higher in the Pris + AKG group (12/14). For the analyses, 6 valid surviving mice and 6 surviving animals in other three groups were randomly selected using a random-number generator, without reference to disease severity or phenotype. All biochemical, histological, molecular, and immunological analyses were performed on the same randomly selected 6 mice. The number of animals for each analysis is indicated in the corresponding figure legends. The euthanasia standards numbers of experimental animals and number of animals surviving in each experimental group at the end of the study were shown in Supplementary Table S1. At the experimental endpoint, the mice were euthanized, and kidney weight and spleen weight were measured; serum, spleen, and kidney tissues were collected for experimental testing. And the corresponding sample sizes for each analysis are now stated in the figure legends.

### 2.4. Histological Examination

All kidney tissues were carefully collected and immersed in a 4% paraformaldehyde solution after euthanasia. Dehydrated with graded ethanol, tissues were embedded in paraffin. Subsequently, continuous transverse sections (5-μm thick) were prepared for histological assessment. Sections were subjected to routine hematoxylin and eosin (HE; Cat. No. G1076; Servicebio; Wuhan; China) to observe the tissue structure, and Masson’s trichrome staining (Cat. No. G1006; Servicebio; Wuhan; China) was performed to access tissue collagen deposition and fibrosis. The Periodic Acid-Schiff (PAS; Cat. No. G1008; Servicebio; Wuhan; China) staining method was used to examine the integrity of the glomeruli and the structure of the basement membrane.

For the detection of IgG deposition in the kidneys, renal sections were probed with the goat anti-mouse IgG H&L (Alexa Fluor^®^ 555) (Cat. No. A21422; Invitrogen™; Thermo Fisher Scientific; DE, USA) at 1:1000 dilution for 60 min at 37 °C, and glomerular IgG deposition was quantified by Mean fluorescence intensity (MFI). Image acquisition was completed using the Zeiss fully automated slide-scanning system (model Axioscan 7) and Zeiss microscope image-processing software (version 3.5). Quantitative analysis of the images was performed on adjacent sections from 6 mice of each group, and 30 glomeruli were analyzed in each section, using ImageJ software (version 1.52, National Institutes of Health, USA). For all histological and immunofluorescence measurements obtained from multiple glomeruli and tissue sections, values were first averaged within each animal, and the resulting means were used for statistical analysis. The individual animal was therefore treated as the independent experimental unit; n refers to the number of animals, not the number of glomeruli or sections.

A semi-quantitative scoring system was used by two blinded independent pathologists. All histological assessments, including renal histopathological analysis and IgG immunofluorescence quantification, were independently conducted by an observer who remained unaware of the treatment groups, with sections coded using neutral labels prior to evaluation. The codes were broken only after all analyses were completed.

### 2.5. Measurement of Serum Enzymes and Indices

Whole blood samples were allowed to clot at room temperature for 2 h, and then centrifuged at 3000 rpm for 15 min at 4 °C. The supernatant was collected as serum. Serum biochemical parameters, including ALT, AST, ALP, ALB, urea, CREA, LDH, CK, CK-MB, UA, and TP, were determined by commercial-kits testing procedures (Shanghai Kehua Bio-engineering Co., Ltd. Shanghai, China). All assays were performed on an automatic biochemical analyzer (HITACHI 3110; Hitachi High-Tech Diagnostics Co., Ltd. Tokyo, Japan) with a detection wavelength range of 340–800 nm, following the manufacturer’s instructions.

### 2.6. Biochemical Index Test

The levels of AKG, T-AOC, SOD, malondialdehyde (MDA) and the activities of CAT in serum were measured by commercial assay kits testing procedures (AKG: Cat. No. S0323S; T-AOC: Cat. No. S0119; SOD: Cat. No. S0109; MDA: Cat. No. S0131S; Cat. No. S0051; Beyotime; Shanghai, China) according to the manufacturer’s guidance. GSH-Px activity was measured using kits from Nanjing Jiancheng Technology Co., Ltd. (Nanjing, China). The urinary-protein (UP) concentration was determined by commercial kit (Cat. No. BC5655; Solarbio Science and Technology Co., Ltd.; Beijing, China), according to the prescribed protocol. Indexes were detected at corresponding absorbance: AKG (570 nm), T-AOC (405 nm), SOD (560 nm), MDA (532 nm), CAT (520 nm), GSH-Px (412 nm), and UP (560 nm), respectively.

### 2.7. Spleen-Cells Isolation

Spleens were washed with ice-cold 0.1 M PBS (pH 7.4), and the spleens were gently ground using the plunger end of a syringe until no obvious red clumps remained. The ground cell suspension was filtered through a 70 μm cell strainer, and the filtered cells were collected into a centrifuge tube and centrifuged at 300 g for 5 min, after which the supernatant was discarded. The cell pellet was resuspended in red-blood-cell lysis buffer (Cat. No. 00-4333-57; Invitrogen™; Thermo Fisher Scientific; DE, USA) and incubated for lysis at room temperature for 5 min. Lysis was then terminated by the addition of PBS, the cells were collected by a second centrifugation. The cell counts were recorded before and after RBC lysis.

### 2.8. Flow-Cytometry Analysis

The splenocytes collected in the previous step were diluted to a concentration of 10^6^ cells/mL with PBS supplemented with 0.5% BSA, then stained with anti-mouse CD45 (APC R660), anti-mouse CD3 (FITC), and anti-mouse CD19 (APC/Fire™ 750) antibodies (APC anti-mouse CD45, Cat. No. 103112; FITC anti-mouse CD3, Cat. No. 100204; APC/Fire™750 anti-mouse CD19, Cat. No. 115558; BioLegend; San Diego; CA; USA). For staining, cells were co-incubated with the antibodies at 4 °C for 30 min in complete darkness, followed by washing with PBS before analysis via flow cytometry. Neither counting beads nor volumetric measurements were used, and absolute cell counts were not derived from total splenocyte numbers. No absolute cell numbers were determined in this study. Consequently, all flow cytometric data are now reported exclusively as relative frequencies. The complete gating strategy is provided in Supplementary Figure S1. Cells were first gated on the FSC-A/SSC-A profile to exclude debris, followed by FSC-A/FSC-H gating to exclude doublets. CD45^+^ leukocytes were then identified within the single-cell population, and CD19^+^ B cells and CD3^+^ T cells were subsequently analyzed within the CD45^+^ gate. Both CD19^+^ B cells and CD3^+^ T cells were quantified as percentages of CD45^+^ leukocytes We would like to clarify that no viability dye was included, and thus nonviable cells were not excluded by a dedicated viability marker; morphology-based gating was applied to minimize the contribution of dead cells and debris. Samples were acquired on a Flow cytometry analyzer (Model: CytoFLEX LX, Beckman Coulter; Brea; CA; USA) and all FACS data were processed using Flowjo (version 10.8.1) software.

### 2.9. Immune-Factor Assay

Levels of antinuclear antibodies (ANAs) and anti-dsDNA antibodies in serum, and the inflammatory factors CXCL10, IL-1β, TNFα, and IL-6 in serum were measured respectively. The ANA ELISA kit (Cat. No. EM1607) and anti-dsDNA kit (Cat. No. EM1471) were sourced from Fine Biotechnology Co., Ltd. (Wuhan, China). The CXCL10 ELISA kit (Cat. No. RK00056) was purchased from ABclonal Biotech Co., Ltd. (Wuhan, China), and the mouse IL-1β (Cat. No. EMC001b.96), TNFα (Cat. No. EMC102a.96) and IL-6 (Cat. No. EMC004.96) kits were purchased from NeoBioscience Technology Co., Ltd. (Shenzhen, China).

### 2.10. Mitochondrial DNA (mtDNA) Copy Number

Total genomic and mitochondrial DNA was extracted from the mouse kidney using a commercial DNA isolation kit (Cat. No. D133-01; GenStar; Beijing; China) according to the manufacturer’s protocols. The relative mtDNA copy number was quantified via quantitative real-time PCR (qPCR) performed using Vazyme SYBR Green PCR Master Mix (Cat. No. Q711-02). Two distinct regions of the mitochondrial genome, namely *mt-Nd1* and *mt-Dloop*, were amplified to represent the mtDNA abundance, while the nuclear *Actb* (encoding β-actin) gene served as the single-copy nuclear reference gene. The relative abundance of mtDNA copy number was calculated as the level relative to the control group. The primer sequences of this research were listed in Supplementary Table S2.

### 2.11. RNA Extraction and Real-Time Quantitative PCR (RT-qPCR)

Total RNA was isolated from kidney tissue with TRIzol reagent (Cat. No. 15596026; Thermo Fisher Scientific; DE, USA) followed the protocols, and purity was checked via 260/280 nm absorbance on a NanoDrop spectrophotometer (Thermo Scientific). A total of 1 μg RNA was reverse-transcribed to cDNA using a Vazyme reverse transcription kit (Cat. No. FT301). RT-qPCR was performed with Vazyme SYBR Green Master Mix on an ABI 7500 Real-Time PCR System (Thermo Fisher Scientific). Cycling steps were 95 °C for 5 min, 40 cycles of 95 °C for 10 s and 60 °C for 30 s, and all reactions were run in three replicates. Primer specificity was assessed by melting curve analysis; a single sharp peak confirmed satisfactory specificity (Supplementary Figure S2). The amplification efficiency (E) of RT-qPCR under these conditions was also evaluated, and all E values fell within 90–110% (Supplementary Figure S3) [31,32]. Relative expression levels were assessed using the 2^−ΔΔCt^ method, with results normalized to the reference gene *Actb*. Primer sequences were shown in Supplemental Table S2.

### 2.12. ATP and ROS Levels in Mouse Kidney

ATP levels in mouse-kidney tissues were measured with an Enhanced ATP Assay Kit (Cat. No. S0027; Beyotime; Shanghai, China). Briefly, we first added the ATP working solution to the test wells to eliminate background, then mixed it with the supernatant from tissue lysis for 2 min. Finally, the RLU value was measured with a spectrophotometer. ROS content in mouse-kidney tissues was measured using a DCFH-DA fluorescent probe kit (Cat. No. S0033S; Beyotime; Shanghai, China), and all operations were carried out exactly following the manufacturer’s protocols. The DCFH-DA probe was incubated with kidney-tissue lysate at 37 °C for 30 min, then washed three times with PBS. The solution was loaded onto a fluorescence microplate for quantitative measurement.

### 2.13. Statistical Analysis

All data generated in this study are expressed as the mean ± standard deviation (SD), and all statistical analyses were performed using GraphPad Prism 10 software (GraphPad Software, San Diego, CA, USA). Except survival and longitudinal body-weight data, group comparisons were analyzed by one-way ANOVA with Tukey’s post hoc test. Followed by Tukey’s post hoc test, one-way analysis of variance (ANOVA) was used to analyze comparisons involving more than two groups, with error rate at α = 0.05. For the two-factor experimental design (Pristane × AKG), two-way ANOVA was performed to evaluate the main effects and interactions for kidney weight, autoantibody levels and quantitative assessment of renal histopathological injuries. A *p*-value < 0.05 was recognized statistically significant. Kaplan–Meier survival curves were constructed for the 16-week study period, with inter-group comparison by the Log-rank (Mantel-Cox) test. Body weight (recorded every month from month 5 to month 8) was analyzed by a linear mixed-effects (LME) model, the linear mixed-effects model was fitted as follows: fixed effects: treatment group, time, and their interaction; random effects: a random intercept for each animal (1/mice), accounting for repeated measures within the same mouse. Missing longitudinal observations resulting from animal mortality were handled by restricted maximum likelihood (REML) estimation, which is valid under the missing-at-random (MAR) assumption and does not require deletion of animals with incomplete records. In all analyses, a *p*-value of less than 0.05 was predefined as the threshold for statistical significance. * *p* < 0.05, ** *p* < 0.01, *** *p* < 0.001.

## 3. Results

### 3.1. AKG Prevents the Development of SLE

**Figure 1:**
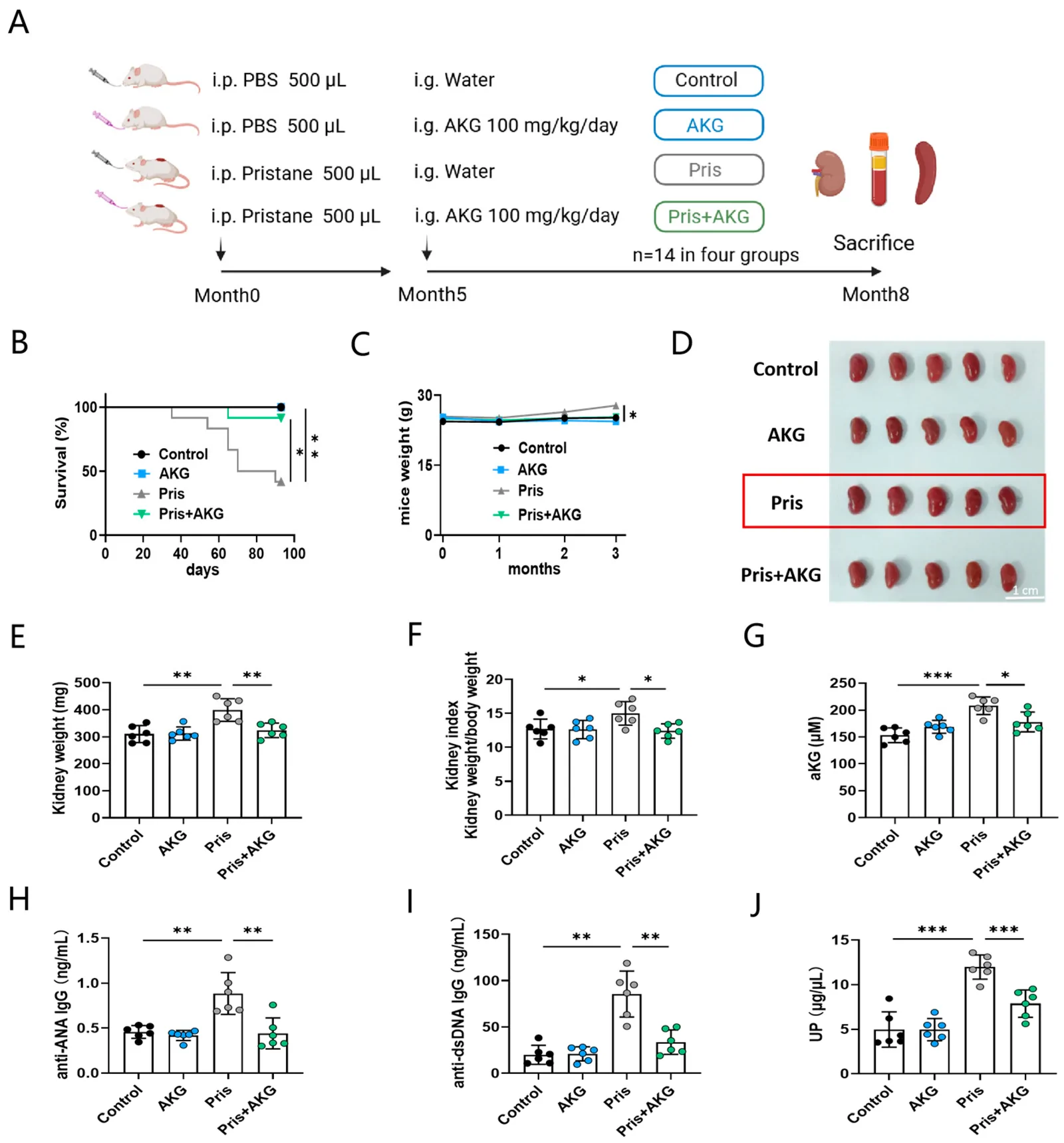
AKG protects suppressed pristane-induced SLE. (**A**) Schematic diagram of the experimental design and α-ketoglutarate (AKG) intervention in a pristane-induced SLE mouse model (*n* = 14); (**B**) Survival curves of mice among the four groups (*n* = 14); (**C**) Mice body-weight changes across the four groups (*n* = 6). (**D**–**F**) Representative images of kidney size, scale bar is 1 cm (**D**), quantitative analysis of absolute kidney weight (**E**), and the calculated kidney-to-body weight ratio (**F**) across the four experimental groups. (**G**) Serum AKG levels in different groups of mice (*n* = 6). (**H**) Measurement of serum anti-nuclear antibody (ANA) levels across the indicated groups (*n* = 6). (**I**) Detection of serum anti-dsDNA antibody levels across the indicated groups (*n* = 6). (**J**) Quantitative analysis of urinary protein content across the four experimental groups (*n* = 6). Data were presented as mean ± standard deviation (SD). One-way ANOVA was applied for statistical analyses. Survival curves were analyzed by the Log-rank (Mantel–Cox) test, longitudinal body-weight was analyzed by a linear mixed-effects (LME) model. Two-way ANOVA was employed to examine the interaction effect of AKG and Pristane: significant interaction for (**F**): F = 8.902, *p* = 0.0073; (**H**): F = 10.62, *p* = 0.0039; (**I**): F = 17.7, *p* = 0.0004; and (**J**): F = 10.40, *p* = 0.0042. Pristane: main effects for (**F**): F = 15.05, *p* = 0.0009; (**H**): F = 13.27, *p* = 0.0016; (**I**): F = 38.54, *p* < 0.0001; and (**J**): F = 61.11, *p* < 0.0001. AKG: main effects for (**F**): F = 7.936, *p* = 0.0106; (**H**): F = 15.27, *p* = 0.0009; (**I**): F = 16.33, *p* = 0.0006; and (**J**): F = 10.47, *p* = 0.0041. The main effects of Pristane and AKG across kidney weight, anti-ANA IgG, anti-ds DNA IgG and UP were significant. * *p* < 0.05; ** *p* < 0.01; *** *p* < 0.001.

To explore the function and mechanism of AKG on pristane-induced systemic lupus erythematosus (SLE). Female BALB/c mice were subjected to a single intraperitoneal injection of pristane, and serum was collected for subsequent biochemical and immunological detection at 5 months post-injection. The results suggested that, compared with the PBS control group, the serum levels of anti-ANA antibodies, anti-dsDNA antibodies, UREA, and CREA, as well as the level of urinary protein in the urine were significantly increased in the pristane group (Supplementary Figure S4A–E), indicating that the SLE model was constructed successfully. Then mice were assigned to four groups randomly: PBS and water (Control), PBS and AKG (AKG), pristane and water (Pris), and pristane and AKG (Pris + AKG) (Figure 1A). Pristane-injected mice (Pris group) showed significantly increased mortality, whereas AKG treatment reduced the mortality rate (Figure 1B). We found that pristane injection led to ascites formation and a rise in body weight in the Pris group, whereas AKG administration significantly mitigated these symptoms (Figure 1C). Upon assessing kidney morphology, we observed an abnormal increase in both kidney volume and weight in the Pris group, which was indicative of severe renal edema, while AKG treatment effectively improved the kidney injury (Figure 1D–F). Interestingly, serum AKG levels were abnormally increased in the pristane-induced model group, whereas exogenous AKG administration significantly suppressed this aberrant elevation (Figure 1G). Furthermore, the abnormal elevation of serum anti-nuclear antibody (ANA) and anti-dsDNA antibody levels (Figure 1H,I), coupled with increased urinary protein content (Figure 1J), confirmed the successful establishment of the pristane-induced SLE mouse model. Crucially, treatment with AKG effectively alleviated the SLE-associated pathological damage in mice.

### 3.2. AKG Alleviates the Histological Changes in the Kidney Tissues of Pristane-Induced Mice

**Figure 2:**
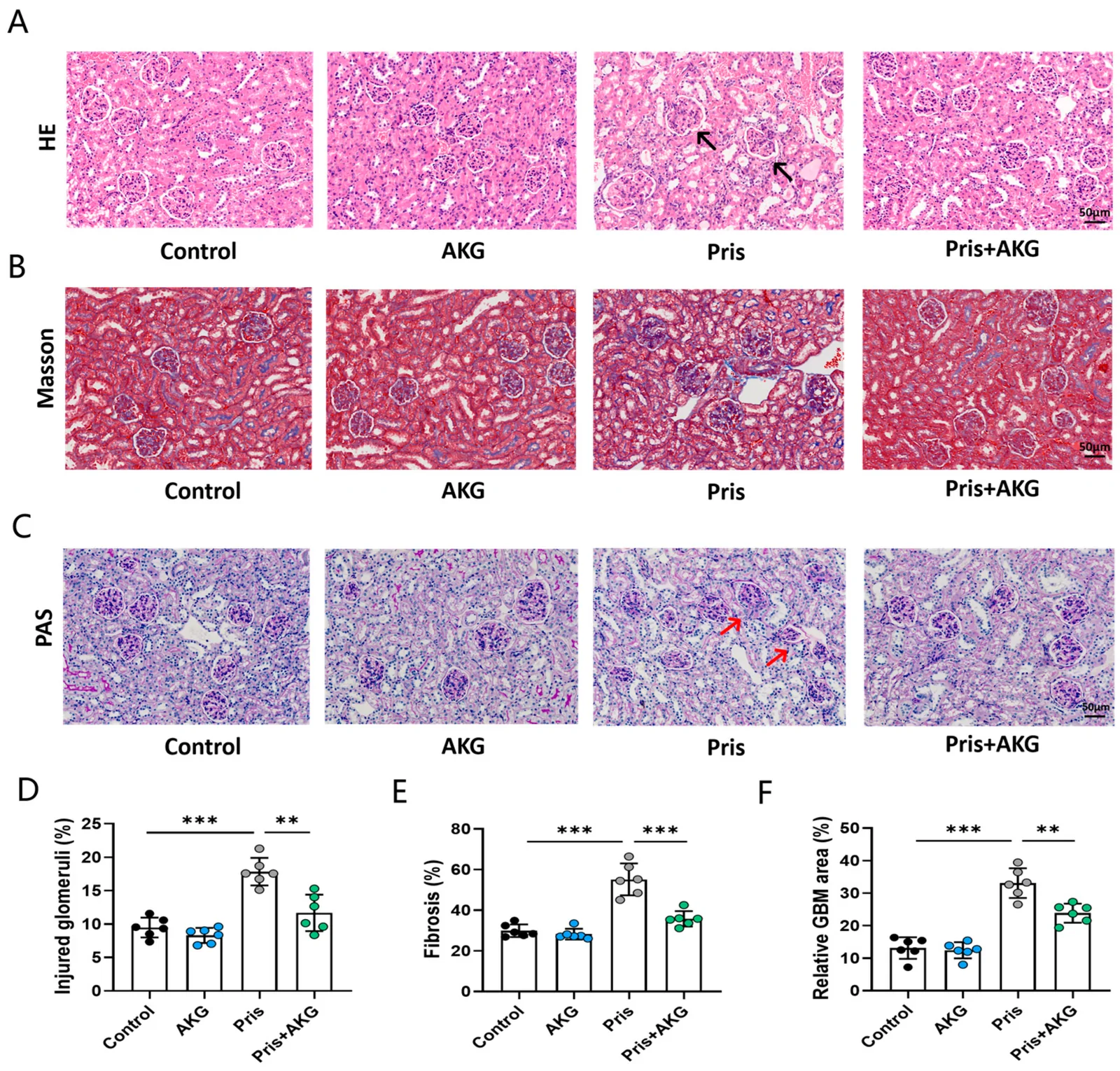
AKG plays a key role in preventing pristane-induced kidney structural damage. (**A**) The kidney histological structures of the Control, AKG, Pris, and Pris + AKG groups observed by H&E staining; arrowheads indicate renal glomerular atrophy. (**B**) The fibrosis in kidney tissues observed by Masson’s trichrome staining. (**C**) The injured glomerulus in kidney tissues determined by PAS staining; arrowheads indicate the thickening of the glomerular basement membrane. (**D**) Percentage of damaged glomeruli across the experimental groups. (**E**) The rate of renal tissue fibrosis. (**F**) The relative glomerular basement-membrane (GBM) area. Each staining experiment and quantitative analysis was repeated in six mice per group (*n* = 6). Data were presented as mean ± standard deviation (SD). One-way ANOVA was applied for statistical analyses. Two-way ANOVA was employed to examine the interaction effect of AKG and Pristane: significant interaction for (**D**): F = 9.773, *p* = 0.0053; (**E**): F = 20.27, *p* = 0.0002 and (**F**): F = 9.318, *p* = 0.0063. Pristane: main effects for (**D**): F = 54.46, *p* < 0.0001; (**E**): F = 69.26, *p* < 0.0001 and (**F**): F = 126.5, *p* < 0.0001. AKG: main effects for (**D**): F = 21.33, *p* = 0.0002; (**E**): F = 28.62, *p* < 0.0001 and (**F**): F = 12.7, *p* = 0.0019. The main effects of AKG and Pristane for injured glomeruli rate, fibross rate, and relative GBM area were significant. Scale bars: 50 μm. ** *p* < 0.01; *** *p* < 0.001.

Histopathological analysis of HE-stained sections revealed that pristane induction triggered profound structural damage in the kidneys, characterized by glomerular hypertrophy, hypercellularity, and inflammatory-cell infiltration (Figure 2A). Morphometric quantification demonstrated that the percentage of damaged glomeruli was markedly elevated in the Pris group compared to the control group (Figure 2D). Strikingly, AKG administration significantly suppressed the pathological trend, substantially reducing the glomerular injury rate. Parallel investigations utilizing Masson’s trichrome staining were conducted to monitor progressive tissue remodeling. Pristane-treated mice exhibited a severe fibrotic area proportion within the renal interstitium (Figure 2B). Notably, AKG intervention effectively alleviated this chronic fibrotic cascade, yielding a prominent decrease in the percentage of renal fibrosis (Figure 2E). Furthermore, PAS staining was employed to indicate the basement membrane integrity and mesangial matrix expansion (Figure 2C). Pristane induced a severe expansion of the relative glomerular basement membrane (GBM) area proportion (Figure 2F). Intriguingly, AKG supplementation remarkably attenuated the aberrant matrix accumulation, preserving the relative GBM area toward the baseline level. To assess the immune-complex deposition, the immunofluorescence staining was used to evaluate the IgG antibody deposits in the renal of the of the Control, AKG, Pris, and Pris + AKG groups (Supplementary Figure S5A). The IgG comments were qualified by mean fluorescence intensity (MFI), which showed that AKG treatment reduced the IgG deposition in the glomeruli caused by pristane (Supplementary Figure S5B). Collectively, these multi-dimensional histological data provide solid evidence that AKG treatment successfully mitigates histopathological damage and prevents structural deterioration in the kidney of SLE mice.

### 3.3. AKG Suppresses Biochemical Alteration in Pristane-Induced SLE Mice

**Figure 3:**
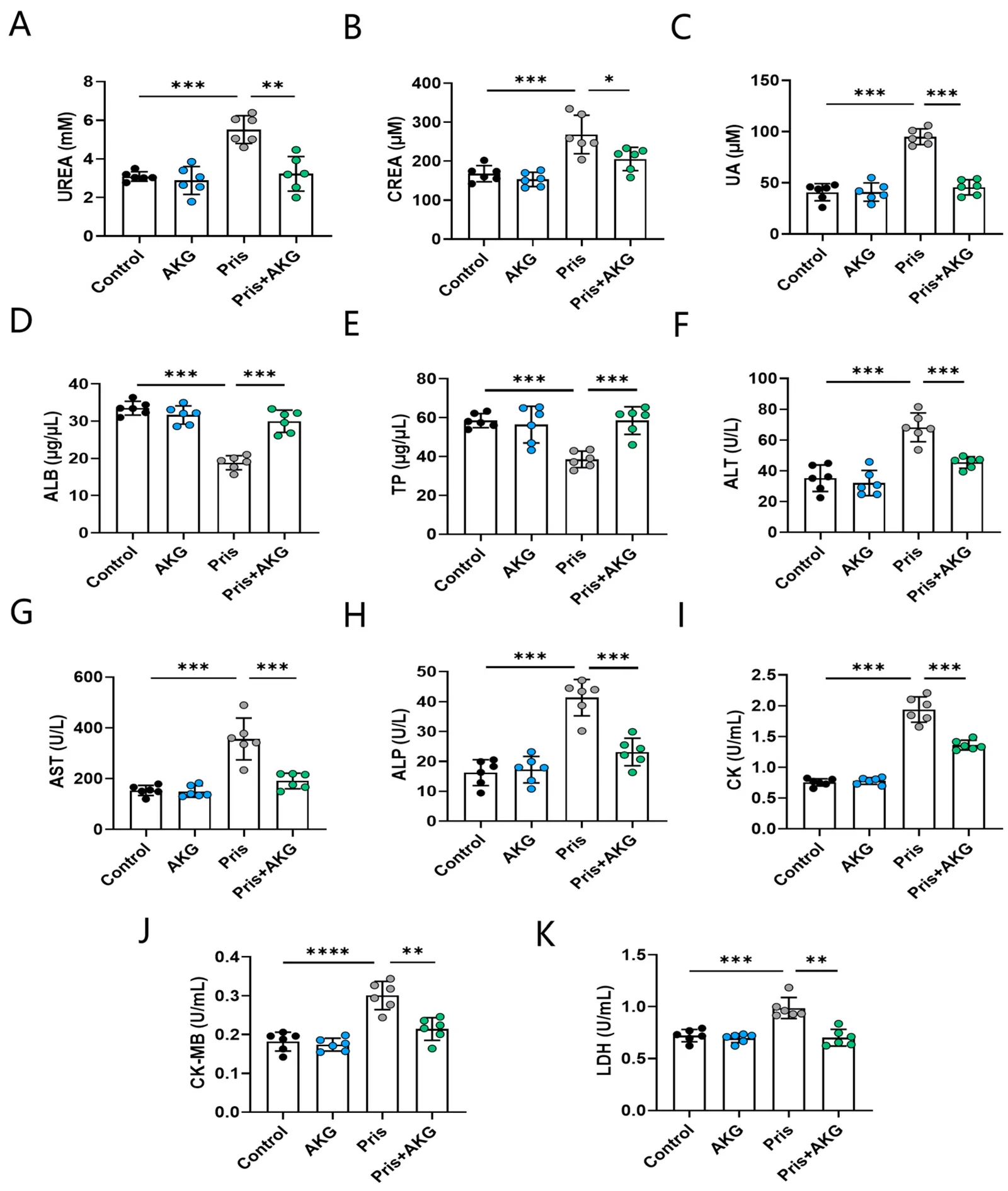
AKG significantly ameliorates multi-tissue functional damage induced by pristane. Serum levels of UREA (**A**); CREA (**B**); Serum levels of UA (**C**); ALB (**D**); TP (**E**); ALT (**F**); AST (**G**); ALP (**H**); CK (**I**); CK-MB (**J**); LDH (**K**). Repeating *n* = 6 in each group. Data were presented as mean ± SD. One-way ANOVA with Tukey’s post hoc test was applied for statistical analyses. * *p* < 0.05, ** *p* < 0.01, *** *p* < 0.001, **** *p* < 0.0001.

Given that systemic lupus erythematosus (SLE) is a prototypical multi-systemic autoimmune disease driven by chronic inflammation [15,33], we further investigated the effect of AKG on systemic biochemical involvement by profiling a comprehensive panel of serum biochemical parameters. We first focused on renal filtration function, the core pathological hallmark of lupus nephritis. Pristane-induced SLE mice exhibited pronounced kidney impairment, characterized by elevations in serum levels of urea (Figure 3A), creatinine (CREA) (Figure 3B), and uric acid (UA) (Figure 3C). Remarkably, AKG significantly reversed the aberrant accumulation, demonstrating its protective effects on glomerular filtration capacity. Additionally, serum albumin (ALB) (Figure 3D) and total protein (TP) (Figure 3E) levels were decreased in the pristane model group. In addition, the transaminases (ALT, AST) and alkaline phosphatase (ALP) concentrations, were markedly upregulated in the pristane model group (Figure 3F–H). These indices reflect that pristane induced severe filtration dysfunction and potentially systemic injuries in mice. Furthermore, we monitored the complement of muscle injury–related enzymes. Pristane triggered a conspicuous surge in serum creatine kinase (CK) (Figure 3I), creatine kinase-MB (CK-MB) (Figure 3J), and lactate dehydrogenase (LDH) (Figure 3K) activities, indicating musculoskeletal inflammation and muscle function impairment in SLE model mice. Strikingly, AKG treatment effectively suppressed the increased levels of these enzymes. Collectively, biochemical analysis provides convergent evidence that AKG intervention exerts broad systemic protective effects, successfully rescuing biochemical alteration in pristane-induced SLE mice.

### 3.4. AKG Has a Significant Effect on Reducing Oxidative Stress in SLE Mice

**Figure 4:**
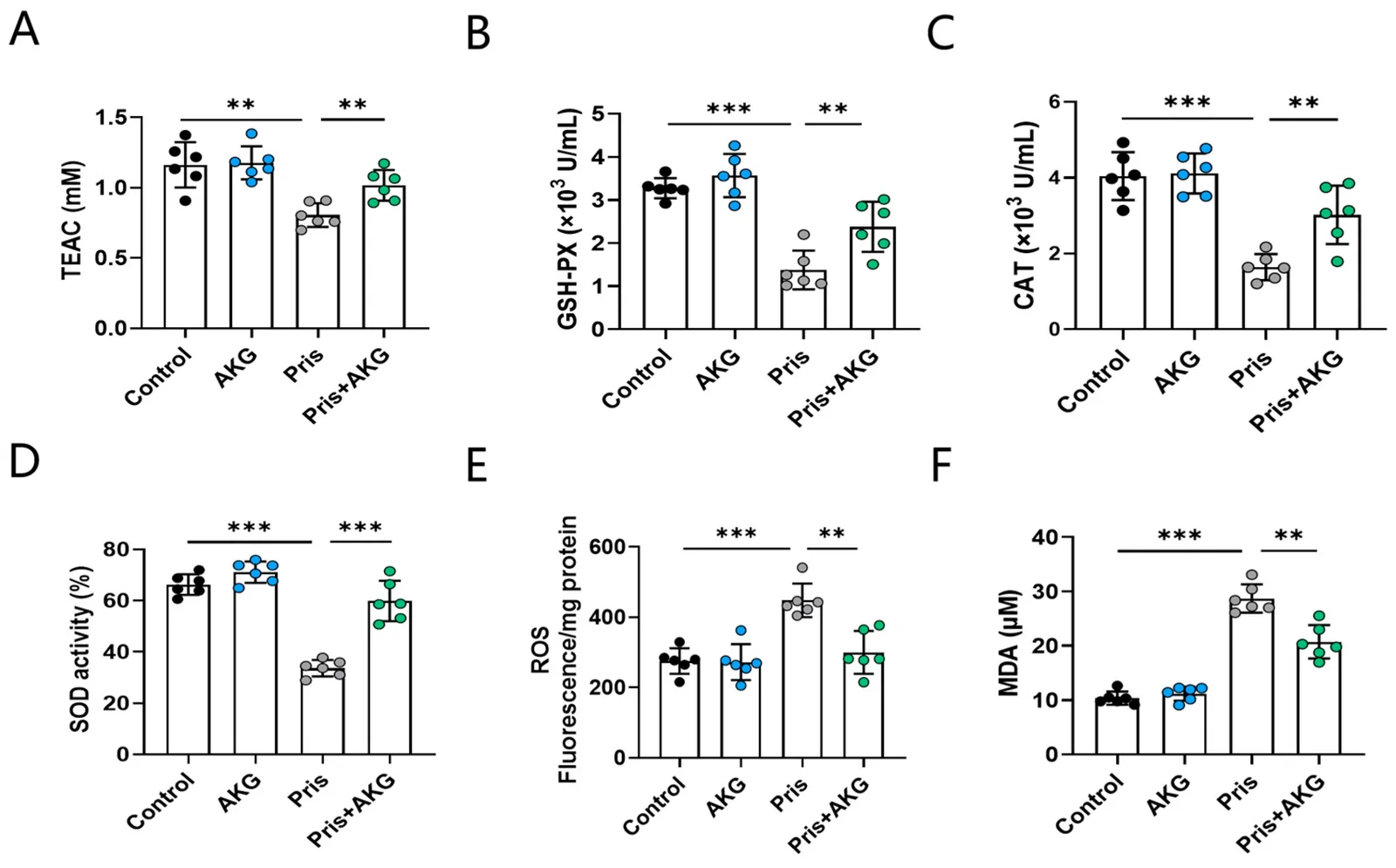
AKG inhibited pristane-induced oxidative stress. (**A**–**D**) Serum levels of TEAC (**A**), GSH-PX (**B**), CAT (**C**), and SOD activity (**D**) in different groups of mice (*n* = 6). (**E**) Levels of ROS in the mice kidney tissues. (**F**) Levels of MDA in the mice serum (*n* = 6). Data were presented as mean ± SD. One-way ANOVA was applied for statistical analyses. ** *p* < 0.01, *** *p* < 0.001.

Previous studies have shown that AKG exerts both antioxidant activity and organ-protective effects [26,34]. To evaluate the efficacy of AKG in counteracting pristane-caused oxidative stress, we determined the capacity of antioxidant enzymes and measured the contents of antioxidant markers in serum and the kidney tissues of pristane-treated mice. Pristane markedly decreased the total antioxidant capacity (T-AOC), as manifested by a profound reduction in total antioxidant capacity (TEAC) (Figure 4A), accompanied with decline in the activities of core antioxidant enzymes, including glutathione peroxidase (GSH-Px) (Figure 4B), catalase (CAT) (Figure 4C), and superoxide dismutase (SOD) (Figure 4D). Conversely, AKG rescued the activities of these key antioxidant effectors, demonstrating its potent capacity to reinforce the antioxidant stress. More critically, AKG administration successfully decreased the ROS (Figure 4E) overload in renal tissue and suppressed the level of serum MDA (Figure 4F). These data justified that AKG treatment effectively protected against pristane-induced oxidative stress.

### 3.5. AKG Regulate Gene Expression of Respiration Complex in SLE

**Figure 5:**
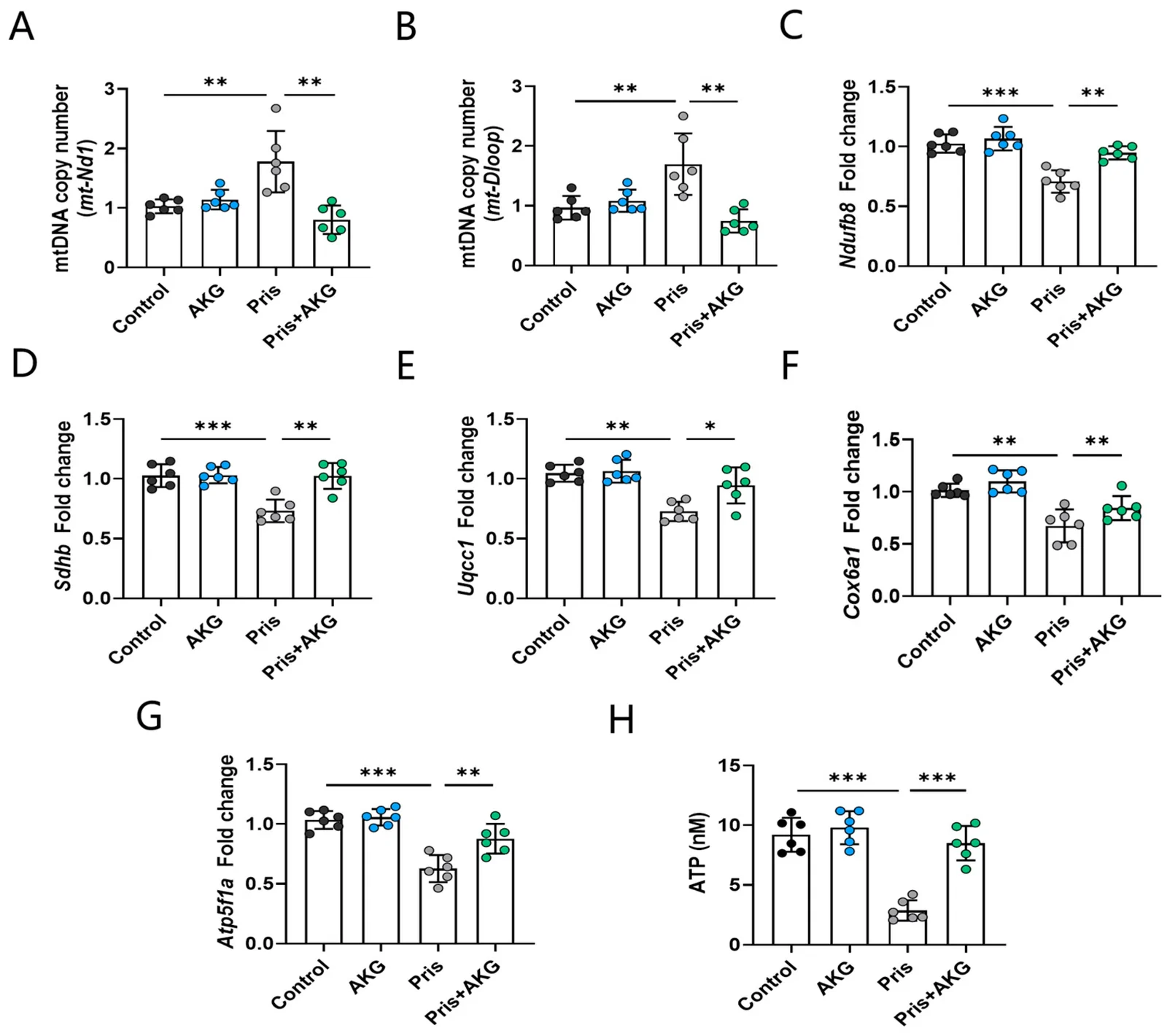
AKG reduces pristane-induced kidney injury by preventing mitochondrial function impairment. (**A**,**B**) The mitochondrial DNA (mtDNA) copy number determined by *mt-Nd1* (**A**) and *mt-Dloop*. (**B**) Levels relative to *Actb* (*n* = 6). (**C**–**G**) Levels of mitochondrial respiratory chain complex-related genes relative to mRNA expression: *Ndufb8*, *Sdhb*, *Uqcc1*, *Cox6a1,* and *Atp5f1a* (*n* = 6). (**H**) Contents of ATP in the kidney tissues of each mouse (*n* = 6). Data were presented as mean ± SD. One-way ANOVA was applied for statistical analyses. * *p* < 0.05, ** *p* < 0.01, *** *p* < 0.001.

Recent studies indicate that mitochondrial mass and circulating mitochondrial DNA (mtDNA) levels can serve as potential diagnostic biomarkers for SLE [35]. In addition to the oxidative-stress assessment described above, we further tested whether AKG could alleviate mitochondrial functional damage in pristane-induced SLE. We first measured the mitochondrial DNA (mtDNA) copy number using two independent markers, *mt-Nd1* and *mt-Dloop*. Pristane treatment caused a significant increase in mtDNA copy number (Figure 5A,B), consisting with abnormal compensatory mitochondrial proliferation under severe inflammatory stress. AKG treatment corrected this genomic dysregulation, bringing the relative mtDNA copy number back to near the control group.

To confirm whether the abnormality further affects mitochondrial energy production, we also measured the mRNA expression levels of key genes encoding the core subunits of the mitochondrial electron transport chain: *Ndufb8*, *Sdhb*, *Uqcc1*, *Cox6a1,* and *Atp5f1a* [36,37,38]. The quantitative results showed that all these critical ETC subunit genes were significantly downregulated in the kidneys of the Pris group (Figure 5C–G). Whereas AKG treatment significantly upregulated the expression of respiratory chain genes, effectively improving the mitochondrial biogenesis-related electron transport system.

ATP production plays an essential role in cell-energy metabolism status [39,40,41]. Therefore, we detected ATP levels in kidney tissues to assess the energy status. As expected, pristane treatment significantly decreased ATP content in the kidney, suggesting that pristane impaired ATP production ability (Figure 5H). Importantly, administration of AKG successfully reversed this ATP deficit in kidney tissue.

Taken together, these multi-dimensional results clearly demonstrate that AKG protects kidney tissue from pristane-induced injury by maintaining mitochondrial levels, upregulating respiratory chain gene expression, and recovering tissue ATP levels.

### 3.6. AKG Blunts the Abnormal Elevation of Immune Cells Induced by Pristane to Alleviate Splenic Injury in SLE Mice

**Figure 6:**
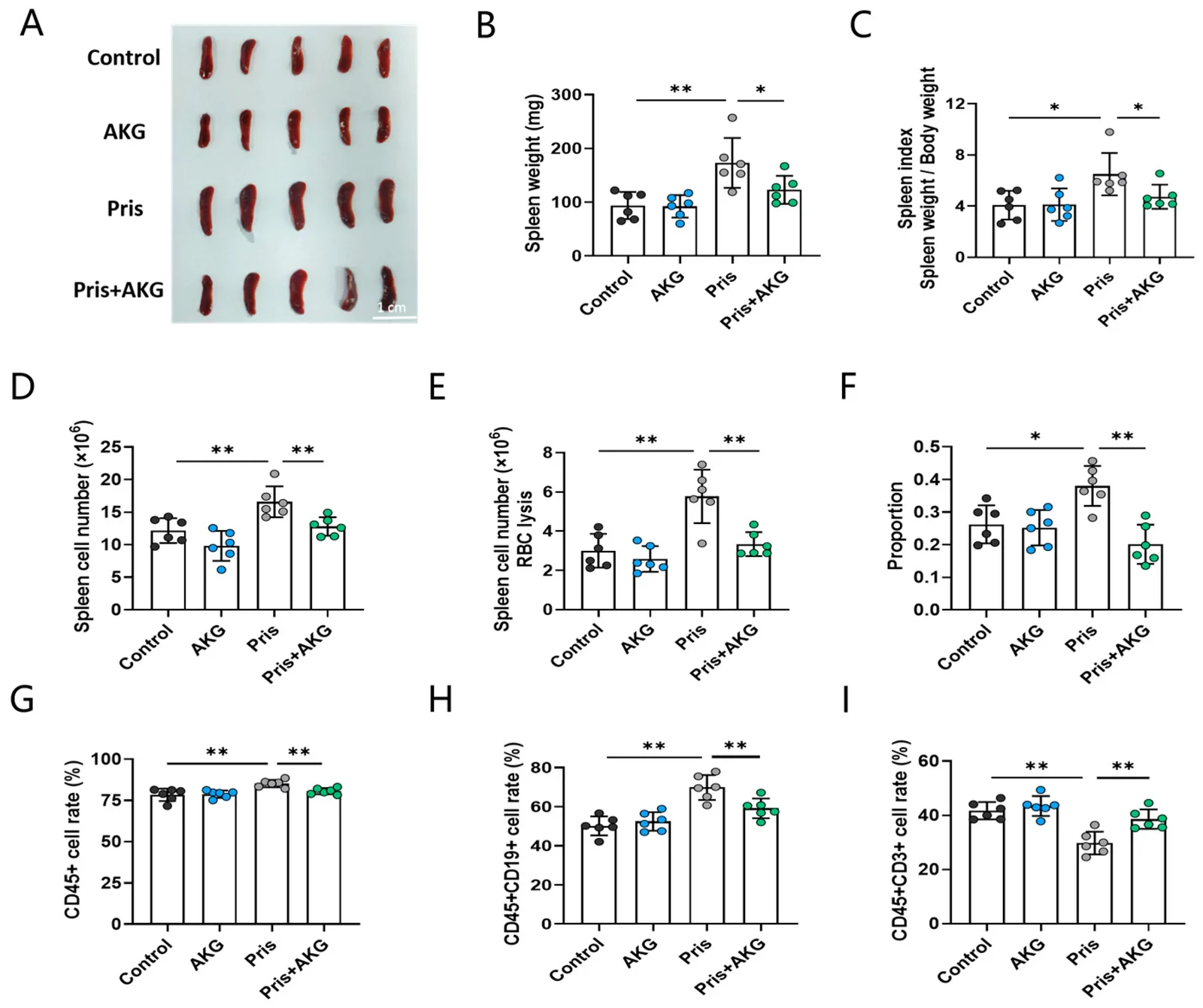
AKG treatment significantly ameliorates the abnormal increase of immune cells in the spleen of SLE mice. (**A**–**C**) Representative images of spleen size, scale bar is 1 cm (**A**), quantitative analysis of absolute spleen weight (**B**), and the calculated spleen-to-body weight ratio (**C**) across the four experimental groups. (**D**) Total number of splenocytes harvested from the four experimental groups (*n* = 6). (**E**) Absolute number of splenocytes obtained after red-blood-cell (RBC) lysis (*n* = 6). (**F**) Percentage of remaining splenocytes after RBC lysis relative to the pre-lysis total cell count (*n* = 6). (**G**) Frequency of CD45^+^ cells (*n* = 6). (**H**) Frequency of CD45^+^ CD19^+^ B lymphocytes (*n* = 6). (**I**) Frequency of CD45^+^ CD3^+^ T lymphocytes (*n* = 6). Data were presented as mean ± SD. One-way ANOVA was applied for statistical analyses. * *p* < 0.05, ** *p* < 0.01.

SLE is associated with severe splenomegaly and abnormal activation of immune cells. To evaluate the effect of AKG on the spleen, we conducted pathological measurements of the spleens. AKG treatment significantly decreased the aberrant increase in spleen size, weight, and spleen index (spleen weight/body weight ratio) in Pris mice (Figure 6A–C), indicating that AKG rescued pristane-induced splenic injury. Simultaneously, AKG decreased splenocyte number in pristane-injected mice (Figure 6D), including with or without RBC-lysed splenocytes (Figure 6E,F).

For further immune subset characterization, we performed hierarchical gating with surface markers. Flow cytometry data confirmed that the frequency of total splenic leukocytes (CD45^+^ immune cells) was significantly upregulated in pristane-induced mice (Figure 6G); AKG intervention effectively improved these pathological immune alterations. Then, we investigated the impact of AKG on lymphocyte subsets by examining the T and B cell proportions. Flow cytometry analysis revealed a significant decrease in the percentage of CD19^+^ B cell in AKG-administered mice compared to pristane-treated mice (Figure 6H), while the proportion of CD3+ T cells was decreased (Figure 6I). These results requested that exogenous AKG can regulate the aberrant proportion of splenic leukocytes and CD19^+^ B cells, and improved splenic-lymphocyte homeostasis.

### 3.7. AKG Improves SLE by Reducing the Inflammatory Response

**Figure 7:**
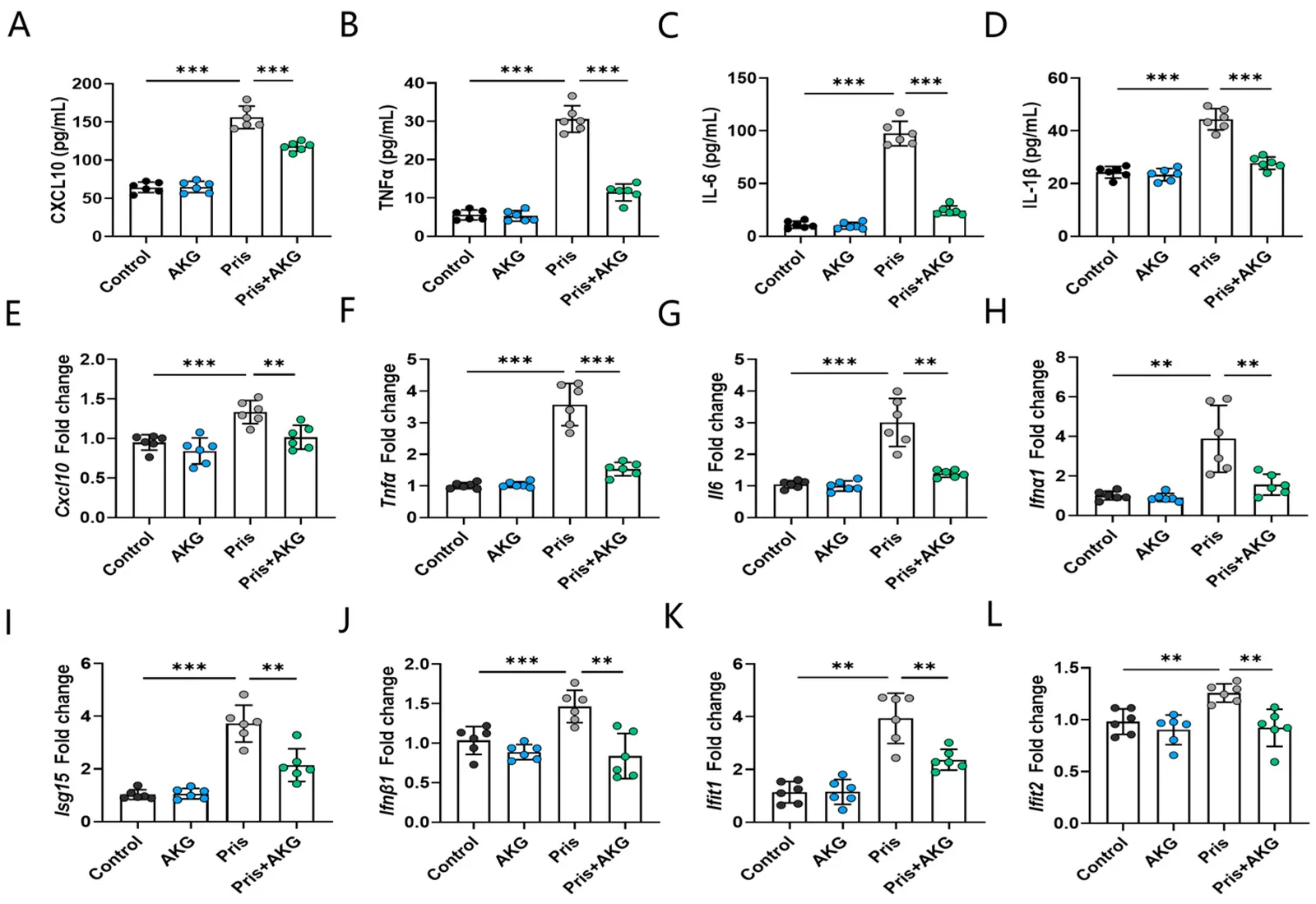
AKG plays an important role in reducing inflammation in pristane-induced SLE mice. (**A**) Measurement of serum CXCL10 levels of all four groups (*n* = 6). (**B**) Detection of serum TNFα levels of all four groups (*n* = 6). (**C**) Detection of serum IL-6 levels of all four groups (*n* = 6). (**D**) Measurement of serum IL-1β levels of all four groups (*n* = 6). (**E**–**G**) Relative mRNA expression levels of inflammatory cytokines: *Cxcl10* (**E**), *Tnfα* (**F**), and *Il6* (**G**) were quantified in four groups (*n* = 6). (**H**–**L**) Quantification of the transcriptional profiles of Type I interferons and downstream ISGs. Relative mRNA expression levels of *Ifna1* (**H**), *Ifnb1* (**I**), *Isg15* (**J**), *Ifit1* (**K**), and *Ifit2* (**L**) across the designated groups (*n* = 6). Data were presented as mean ± SD. One-way ANOVA was applied for statistical analyses. ** *p* < 0.01; *** *p* < 0.001.

To assess the anti-inflammatory effect of AKG, we performed a comprehensive analysis of inflammatory cytokines in serum and interferon-associated genes expression in kidney tissues. Compared with the control group, mice in the Pris group showed significantly elevated serum concentrations of CXCL10, TNFα, IL-6, and IL-1β (Figure 7A–D). AKG administration strongly inhibited the pathological release of these circulating cytokines. Additionally, the expression levels of *Cxcl10*, *Tnfa*, and *Il6* (Figure 7E–G) were all significantly upregulated in the Pris group relative to Control group, while AKG supplement significantly downregulated the transcriptional levels.

Considering that excessive activation of type I interferon serves as a central trigger for inflammatory stress and the pathogenesis of SLE [20,42], we next evaluated the transcriptional expression of the type I interferon signature, the mRNA expression of type I interferon (*Ifna1* and *Ifnb1*) and type I interferon-stimulated genes (*Isg15*, *Ifit1* and *Ifit2*) in the kidney tissue. The mRNA expression levels in Pris group were notably upregulated, while AKG treatment downregulated the transcriptional levels of ISGs (Figure 7H–L).

Collectively, these results provide convincing evidence that AKG ameliorates lupus-like pathogenesis by restraining systemic inflammatory cascades and inhibiting the aberrant type I interferon response.

## 4. Discussion

Systemic lupus erythematosus (SLE) is a heterogeneous, multifaceted, chronic autoimmune disease with multiple organ injuries [1]. Accumulating evidence has demonstrated that SLE progression is driven by diverse regulatory factors, encompassing genetic predisposition, mitochondrial functional dysregulation, epigenetic modifications, hormonal modulation, and environmental triggers [43]. SLE is a heterogeneous disease occurring frequently among women and is least common among children. SLE most commonly afflicts women between puberty and menopause, and the female/male ratio shifts from 3/1 in children to about 9/1 or even 15/1 among adults between puberty and menopause [9,44]. Mitochondria have been described as key players in SLE, and targeting these organelles may be a worthwhile therapeutic option [10]. As a key metabolite in the tricarboxylic-acid (TCA) cycle, α-ketoglutarate (AKG) possesses anti-immune, antioxidant, and metabolic regulatory properties [34,45,46]. Hence, this study aims to investigate the protective effects of AKG on female SLE.

In the present research, we used intraperitoneal injection of pristane to establish the SLE model and then investigated the therapeutic effects of AKG on SLE. Firstly, we successfully established a severe, classically validated SLE model. Our data indicate that 3 months of AKG intervention initiated at the established stage of pristane-induced lupus model shows potential as a therapeutic adjuvant for established lupus nephritis, a pathological phenotype characterized by significantly elevated serum antinuclear antibody (ANA) concentrations, anti-dsDNA antibody levels, and severe injuries including the kidney, spleen, and liver [2,47]. Then, we used multi-modal pathological assessment combining hematoxylin–eosin (HE), periodic acid-Schiff (PAS), and Masson’s trichrome staining, which collectively demonstrated that pristane triggered glomerular structural disruption and progressive fibrotic remodeling. This histological damage directly impaired glomerular filtration function, as corroborated by concurrent clinical surges in serum urea, creatinine, and uric acid levels [8,16]. Moreover, the immunofluorescence images show that AKG intervention can significantly reduce the IgG deposition in the renal glomeruli caused by pristane. Additionally, the elevations of hepatic aminotransferases (ALT, AST, ALP) and myocardial injury enzymes (CK, CK-MB, LDH) confirmed the widespread, multi-system injuries of pristane-induced SLE [47,48]. Finally, oral administration of AKG at 100 mg/kg/day remarkably mitigated these diffuse pathological injuries, restoring both tissue architectural integrity and circulating biochemical markers. Hence, we confirmed the protective capacity of AKG in the SLE model. AKG treatment in control mice did not cause any observable adverse effects, highlighting its potential. Future trials should verify the optimize the dosage and preclinical potential.

In this study, mortality was confined mainly to the Pris group, whereas the Pris + AKG groups showed markedly higher survival. Because the intervention spanned three months and pristane-induced lupus is progressive, the deaths observed were consistent with late-stage disease progression [2,49]. Additionally, mice in the Pris + AKG group also died, indicating that mortality reflected disease severity rather than any selective retention of mildly affected animals. Importantly, the improved survival in the AKG-treated groups itself represents a principal therapeutic readout, suggesting that AKG attenuated disease severity. We should acknowledge that survival bias cannot be entirely excluded, the survival pattern supports rather than undermines our conclusion. In addition, we should acknowledge that we just analyzed 6 mice in each group; therefore, further analyses of these samples would strengthen the conclusions in future work.

Serum AKG levels were found to be abnormally elevated in inflammatory diseases, consistent with previous studies [24,27,50]. This is likely due to an impaired TCA cycle or reduced turnover efficiency during periods of active autoimmunity, leading to aberrant AKG accumulation. Interestingly, after exogenous AKG intervention, the intracellular levels of this metabolite were significantly decreased. This observation suggests a negative feedback regulatory mechanism: exogenous AKG may enhance overall metabolic efficiency by reducing inflammation or improving mitochondrial function, thereby promoting the clearance of endogenous accumulations. However, it is clearly stated that the mechanism underlying this observation remains to be established in future studies.

Pathological redox imbalance and mitochondrial impairment are central drivers of SLE progression [35,51]. Here, we found that exogenous AKG treatment effectively improved this self-perpetuating vicious cycle by upregulating endogenous antioxidant enzymes (SOD, CAT, GSH-Px) while significantly neutralizing the fulminant local ROS burst and attenuating lipid peroxidation (MDA) [52].

Oxidative stress acts as both an upstream trigger of SLE and important mediator of mitochondrial injury [10,53]. Then, we explored the effects of AKG on mitochondrial quantification; we performed mtDNA copy number analysis in pristane-treated mice. Unexpectedly, pristane treatment led to an abnormal increase in mitochondrial DNA copy number, a phenomenon newly identified in this study. AKG significantly corrected this abnormal mitochondrial DNA copy number, suggesting that the underlying mechanism may involve compensatory synthesis or impaired mitophagy [52,54]. The most critical function of mitochondria is ATP synthesis, which requires intact mitochondrial electron transport chain function [55]. Transcriptional analysis of genes such as *Ndufb8*, *Sdhb*, *Uqcc1*, *Cox6a1,* and *Atp5f1a* revealed that pristane treatment damaged the mitochondrial electron transport chain [36,37,38]. AKG treatment effectively enhanced ATP production capacity in kidney tissue, consisted with upregulated the gene expression of the mitochondrial electron transport chain. Meanwhile, we should acknowledge that the current study provides only indirect evidence, and that direct mitochondrial functional assays like Seahorse analysis will be needed in future work.

Recent studies have demonstrated evidence of widespread mitochondrial dysfunction across multiple immune-cell populations and in SLE patients [52]. B cells, dendritic cells (DCs), and macrophages are crucial cell populations implicated in SLE pathogenesis [56]. Notably, B cells play a central role in disease development by orchestrating antigen presentation, producing cytokines, and differentiating into antibody-secreting plasma cells, thereby contributing substantially the disease progression [57,58]. In this study, flow-cytometric analysis demonstrated that AKG administration significantly improved immune response and regulated the abundance of immune cells, effectively constraining the aberrant expansion of CD45^+^ splenic leukocytes and CD19^+^ B cell populations, aligning with findings by Gao, Y. et al. [24]. Additionally, we demonstrated that AKG alleviates SLE by preventing oxidative stress and reducing mitochondrial function damage. Of note, the reduced percentage of CD3^+^ T cells observed in the AKG group arose as a secondary “denominator dilution effect” driven by the massive numerical burst of B cells [59]. By curbing pathological CD19^+^ B cell abundance, AKG improved physiological lymphocyte subset distribution, a phenotypic change that directly correlated with reduced levels of circulating pro-inflammatory cytokines, including CXCL10, TNFα, and IL-6, and decreased the upregulated expression of type I interferon signature induced by pristane [2,20].

Although we have clearly demonstrated the benefits of AKG at both tissue and systemic levels in SLE, some limitations should be acknowledged. For example, the precise molecular targets of AKG within specific immune-cell subsets and the underlying mechanisms remain to be elucidated. Immune-cell subsets are reported as percentages of the gated populations, absolute cell numbers are not reported. In addition, viability dye was not used, so nonviable cells could not be excluded. Although B-cell differentiation and antibody-secreting cells are central to SLE pathogenesis, we did not perform deeper phenotyping of B-cell subsets, such as activated B cells and plasmablast/plasma cells. Further characterization of these immune-cell subsets would be valuable in future studies. Regardless of whether AKG regulates B-cell differentiation through epigenetic histone demethylases, cell surface receptors, or other mechanisms, the exact underlying mechanism still requires further detailed investigation. The current study was limited to a single-dose intervention in a murine SLE model. Future studies should test multiple dose levels to establish the dose-response relationship and enhance the translational potential of these findings. Importantly, clinical translation will require further validation, including dose-response and safety studies before any human application.

## 5. Conclusions

**Figure 8:**
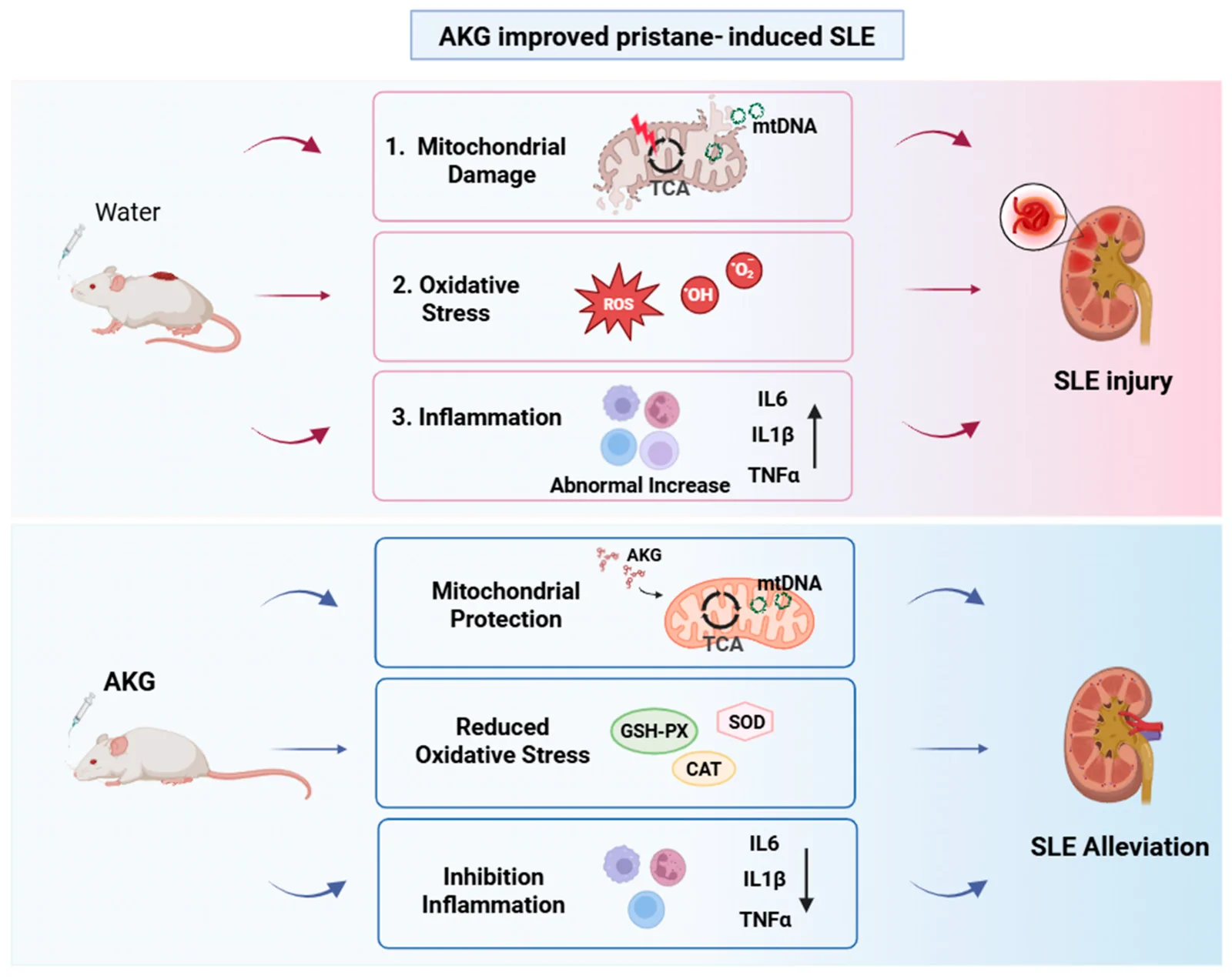
Schematic illustration of AKG against pristane-induced SLE. Intraperitoneal injection of pristane established a systemic lupus erythematosus (SLE) mouse model, with mitochondrial damage, oxidative stress, abnormal proliferation of inflammatory cells, and enhanced inflammatory responses, ultimately leading to impaired renal function. After intervention with α-ketoglutarate (AKG), these pathological injuries were significantly ameliorated, ultimately suppressing the multiple damages associated with systemic lupus erythematosus. Three arrows on the left and right represent three pathways of action. Upward arrows denote upregulation and downward arrows denote downregulation. This figure was created in BioRender (https://BioRender.com).

In summary, our results indicate that AKG could significantly ameliorate pristane-induced SLE by promoting the catalytic activity of antioxidant enzymes, improving mitochondrial biogenesis-related factors, modulating broad CD3^+^ T and CD19^+^ B-cell proportions, and inhibiting inflammatory responses (Figure 8). These findings suggest that AKG improve SLE development by pleiotropic mechanism involving oxidative stress and inflammation.

## Data Availability

Data can be obtained in the article and Supplementary Materials.

## Supplementary Materials

The following supporting information can be downloaded at: https://www.mdpi.com/article/10.3390/nu18213600/s1, Supplementary Table S1: Numbers of experimental animals. Supplementary Table S2: Target genes and primer sequences. Supplemental Figure S1: Gating strategy for flow cytometric analysis of splenocytes. Supplemental Figure S2. Melting Curve Analysis. Supplemental Figure S3. Efficiency analysis. Supplementary Figure S4. Pristane-induced murine model of SLE. Supplementary Figure S5. AKG intervention reduces IgG antibody deposition in glomeruli.

## Abbreviations

The used abbreviations in this manuscript are as following:

AKG	α-ketoglutarate
Pris	Pristane
UP	Urine Protein
GBM	Glomerular Basement Membrane
CREA	Creatinine
UA	Uric Acid
ALB	Albumin
TP	Total Protein
ALT	Alanine Aminotransferase
AST	Aspartate Aminotransferase
ALP	Alkaline Phosphatase
CK	Creatine Kinase
CK-MB	Creatine Kinase-MB
LDH	Lactate Dehydrogenase
TEAC	Trolox-Equivalent Antioxidant Capacity
CAT	Catalase
MDA	Malondialdehyde
*mt-Nd1*	Mitochondrially Encoded NADH Dehydrogenase 1
*mt-Dloop*	Displacement Loop
*Ndufb8*	NADH dehydrogenase (ubiquinone) 1 beta subcomplex, 8
*Sdhb*	Succinate Dehydrogenase Complex Iron-Sulfur Subunit B
*Uqcc1*	Ubiquinol-Cytochrome C Reductase Complex Chaperone 1
*Cox6α1*	Cytochrome C Oxidase Subunit 6A1
*Atp5f1α*	ATP Synthase F1 Subunit Alpha
CXCL10	C-X-C Motif Chemokine Ligand 10
TNFα	Tumor Necrosis Factor Alpha
IL-1β	Interleukin 1 Beta
IL-6	Interleukin 6
*Ifna1*	Interferon Alpha 1
*Ifn**b1*	Interferon Beta 1
*Isg15*	Interferon Stimulated Gene 15
*Ifit1*	Interferon Induced Protein With Tetratricopeptide Repeats 1
*Ifit2*	Interferon Induced Protein With Tetratricopeptide Repeats 2
