# Supplementary material for: α-Ketoglutarate Alleviates the Pathogenesis of Systemic Lupus Erythematosus via Reducing Oxidative Stress and Attenuating Excessive Immune Response
Source: Nutrients. 2026 Oct 30;18(21):3600. doi: 10.3390/nu18213600 (PMC13765487; doi:10.3390/nu18213600)
Supplement: Supplementary file 1 [file nutrients-18-03600-s001.zip › Supplementary Figure.pdf]

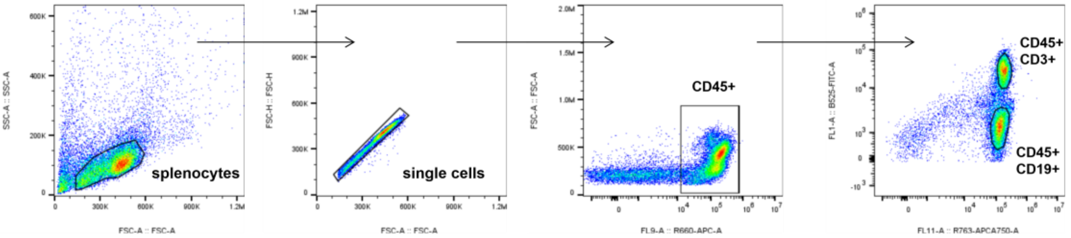

Supplemental Figure S1. Gating strategy for flow cytometric analysis of splenocytes. Cells were first gated on the FSC-A/SSC-A profile to exclude debris, followed by FSC-A/FSC-H gating to exclude doublets. CD45⁺ leukocytes were then identified within the single-cell population, and CD19⁺ B cells and CD3⁺ T cells were analyzed within the CD45⁺ gate. No viability dye was used and representative plots are shown.

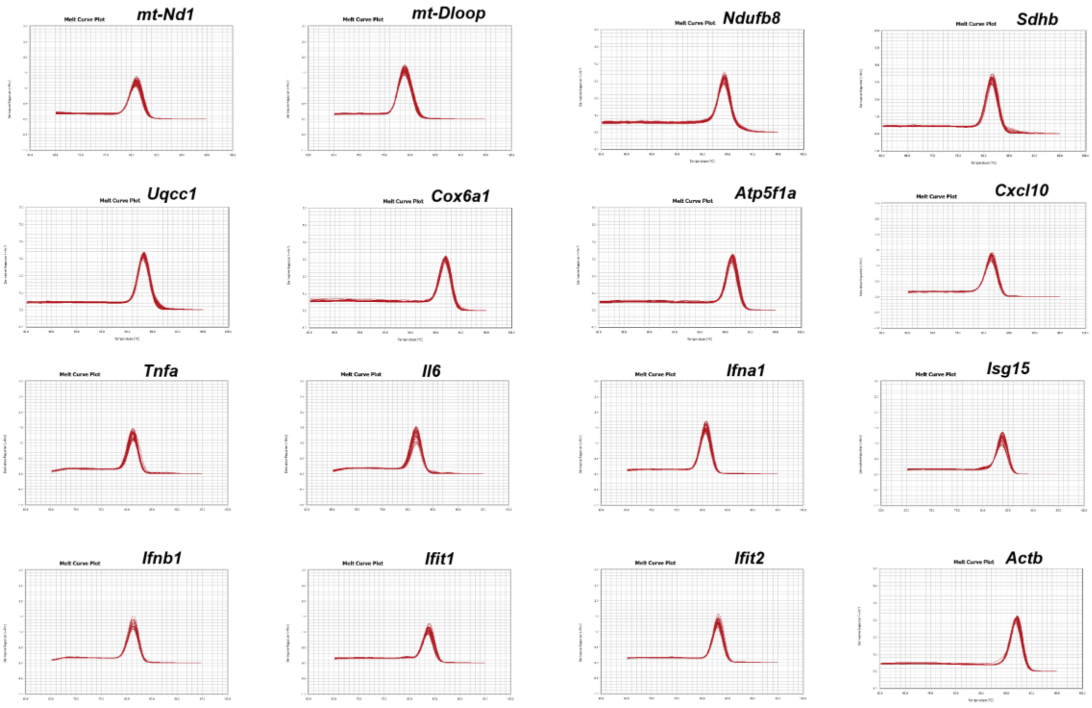

Supplemental Figure S2. Melting Curve Analysis. All primer melting curve peaks are sharp and uniform, with no small peaks in the low-temperature region. This indicates that the primers have excellent specificity, no primer dimers are formed, and there is no non-specific amplification.

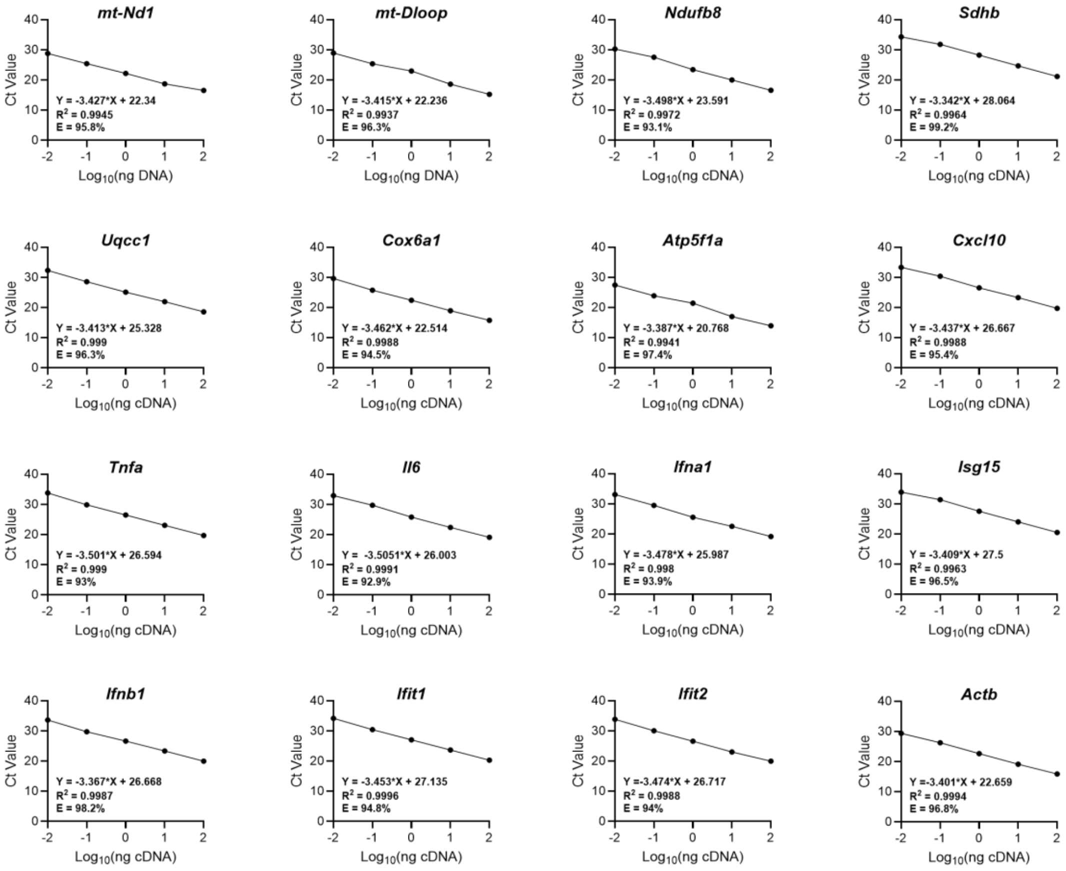

Supplemental Figure S3. Efficiency analysis. The efficiency (E) of all genes ranged from 90% to 110%, indicating good RT-qPCR amplification efficiency.

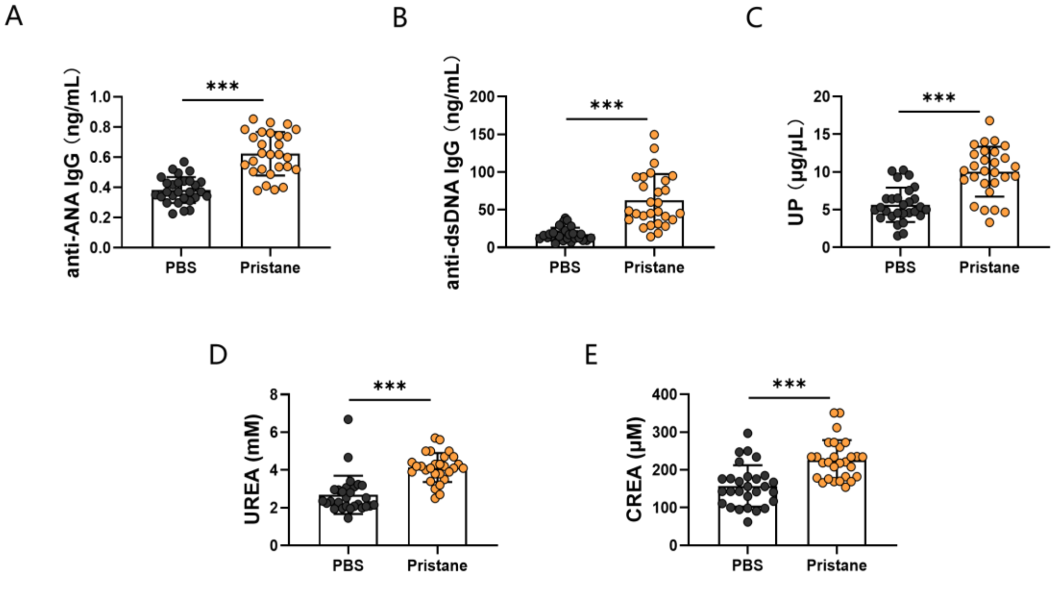

Supplementary Figure S4. Pristane-induced murine model of SLE. (A) Measurement of serum anti-nuclear antibody (ANA) levels of the PBS and Pristane groups (n = 28); (B) Detection of serum anti-dsDNA antibody levels of the PBS and Pristane groups (n = 28). (C) Quantitative analysis of urinary protein content of the PBS and Pristane groups (n = 28). (D) Serum levels of UREA of the PBS and Pristane groups (n = 28). (E) Serum levels of CREA of the PBS and Pristane groups (n = 28). Quantitative

analysis of urinary protein content across the four experimental groups (n = 28). All data were presented as mean ± standard deviation (SD). Statistical analysis was performed with unpaired two-tailed Student's t-test. ***$p < 0.001$.

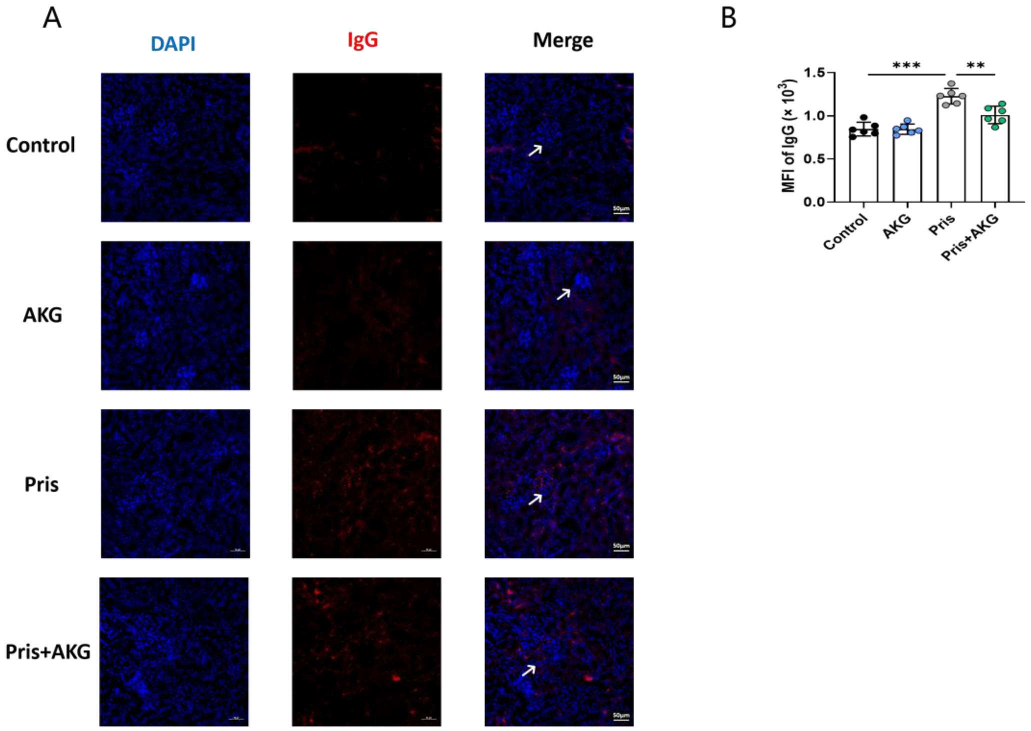

Supplementary Figure S5. AKG intervention reduces IgG antibody deposition in glomeruli. (A) Representative fluorescence imaging (n = 6 images in each group) of IgG (red) and nucleus (blue) in Control, AKG, Pris and Pris+AKG groups. Scale bar, 50 μm. The arrows indicated the IgG antibody deposits in the glomeruli, and the red color indicated the IgG antibodies. (B) The mean fluorescence intensity (MFI) of IgG in the glomeruli (n = 6). Each group had n = 6 mice, and 30 glomeruli were counted for each sample. Statistical analysis was performed by one-way ANOVA with Tukey' s post hoc test. Two-way ANOVA was used to evaluate the interaction between AKG and Pristane: significant interaction for (B): $F(1,20) = 9.951$, p = 0.0050; main effects of Pristane ($p < 0.0001$) and AKG ($p = 0.0049$) was significant ($p = 0.0049$). Scale bars: 50 μm. ** $p < 0.01$; *** $p < 0.001$.
