# Supplementary material for: α-Ketoglutarate Alleviates the Pathogenesis of Systemic Lupus Erythematosus via Reducing Oxidative Stress and Attenuating Excessive Immune Response
Source: Nutrients. 2026 Oct 30;18(21):3600. doi: 10.3390/nu18213600 (PMC13765487; doi:10.3390/nu18213600)
Supplement: Supplementary file 1 [file nutrients-18-03600-s001.zip › Supplementary Table.pdf]

Supplementary Table S1. Numbers of experimental animals.

Group	Assigned (start)	Survived (endpoint)	Analyzed
Control	14	14	6
AKG	14	14	6
Pris	14	6	6
Pris+AKG	14	12	6

Strict pre-defined humane endpoints were implemented for immediate, unplanned early euthanasia:

1. Sustained body weight loss exceeding 20% relative to individual pre-injection baseline
2. Severe lethargy, complete loss of spontaneous movement, or inability to independently access food and water
3. Clinically detectable abdominal distension consistent with massive pristane-induced ascites that impaired normal mobility or respiration
4. Any observable sign of persistent severe pain that could not be alleviated via non-pharmacological welfare intervention.

Animals meeting any of the above criteria were humanely euthanized via CO_2 inhalation followed by cervical dislocation, in strict accordance with institutional IACUC protocol, prior to the onset of irreversible severe distress.

Supplementary Table S2. Target genes and primer sequences.

Gene name	Forward primer Sequence (5'->3')	Reverse Primer Sequence (5'->3')
mt-Nd1	CTAGCAGAAACAAACCGGGC	CCGGCTGCGTATTCTACGTT
mt-Dloop	AGGTTTGGTCCTGGCCTTAT	GTGGCTAGGCAAGGTGTCTT
Ndufb8	CGCCAAGAAGTATAACATGCGAG	CCTCTCATGCTGTGATCGGTTG
Sdhb	TGCGGACCTATGGTGTTGGATG	CCAGAGTATTGCCTCCGTTGATG
Uqcc1	CAGCCTGTTGAAGAGAAGGTCG	CGAAGTCAGTCTTCTCCACGCA
Cox6a1	TCAACGTGTTCCTCAAGTCGC	AGGGTATGGTTACCGTCTCCC
Atp5f1a	TGGTGAAGAGACTGACGGATGC	TCAAAGCGTGCTTGCCGTTGTC
Cxcl10	ATCATCCCTGCGAGCCTATCCT	GACCTTTTTTGGCTAAACGCTTTC
Tnf-α	GGTGCCTATGTCTCAGCCTCTT	GCCATAGAACTGATGAGAGGGAG
Il-6	TGCCACCTTTTGACAGTGATG	CGCACTAGGTTTGCCGAGTA
Ifna1	GGATGTGACCTTCCTCAGACTC	ACCTTCTCCTGCGGGAATCCAA
Ifnb1	GCCTTTGCCATCCAAGAGATGC	ACACTGTCTGCTGGTGGAGTTC
Isg15	CATCCTGGTGAGGAACGAAAGG	CTCAGCCAGAACTGGTCTTCGT
Ifit1	TACAGGCTGGAGTGTGCTGAGA	CTCCACTTTCAGAGCCTTCGCA
Ifit2	CGAACTACCGTCTGGATGACTG	CTTCAACCAGCGCCATTGCTTG
Actb	GGTACCACCATGTACCCAGG	AAACGCAGCTCAGTAACAGTC
